# Communication between Brain Areas Based on Nested Oscillations

**DOI:** 10.1523/ENEURO.0153-16.2017

**Published:** 2017-03-27

**Authors:** Mathilde Bonnefond, Sabine Kastner, Ole Jensen

**Affiliations:** 1 Donders Institute, Centre for Cognitive Neuroimaging, Radboud University, 6525 Nijmegen, Netherlands; 2 Lyon Neuroscience Research Center (CRNL), Brain Dynamics and Cognition Team, INSERM U1028, CNRS UMR5292, Université Claude Bernard Lyon 1, UdL, Lyon, France; 3 Princeton Neuroscience Institute and Department of Psychology, Princeton University, Princeton, New Jersey 08544; 4 University of Birmingham, School of Psychology, Centre for Human Brain Health, Birmingham B15 2TT, UK

**Keywords:** alpha, brain communication, cross-frequency coupling, gamma, slow oscillations, theta

## Abstract

Unraveling how brain regions communicate is crucial for understanding how the brain processes external and internal information. Neuronal oscillations within and across brain regions have been proposed to play a crucial role in this process. Two main hypotheses have been suggested for routing of information based on oscillations, namely communication through coherence and gating by inhibition. Here, we propose a framework unifying these two hypotheses that is based on recent empirical findings. We discuss a theory in which communication between two regions is established by phase synchronization of oscillations at lower frequencies (<25 Hz), which serve as temporal reference frame for information carried by high-frequency activity (>40 Hz). Our framework, consistent with numerous recent empirical findings, posits that cross-frequency interactions are essential for understanding how large-scale cognitive and perceptual networks operate.

## Significance Statement

To understand how the brain operates as a network, it is essential to identify the mechanisms supporting communication between brain regions. Based on recent empirical findings, we propose a mechanism for selective routing based on cross-frequency coupling between slow oscillations in the alpha and gamma bands.

## Introduction

Humans operate in complex environments requiring the encoding and processing of a constant flow of sensory information. While the information must be prioritized, the mechanisms underlying the selective routing of sensory information remain to be understood. Neuronal oscillations, in which excitability is modulated by the phase of the rhythm, have been proposed to play important mechanistic roles for routing information, since they can change the dynamic interactions between brain regions on a fast time scale (Varela et al., 2001). Two hypotheses have been proposed for routing of information based on oscillations (see also Akam and Kullmann, 2014): communication through coherence (CTC; Fries, 2005, 2009, 2016; Bastos et al., 2015) and gating by inhibition (GBI; Jensen and Mazaheri, 2010). The CTC framework, at least in its initial form, mainly focused on gamma activity (>30 Hz) while the GBI is mainly based on alpha oscillations (8–13 Hz). These two frameworks are not mutually exclusive, and the aim of this paper is to unify them.

## The CTC and GBI Frameworks

**Figure 1.**
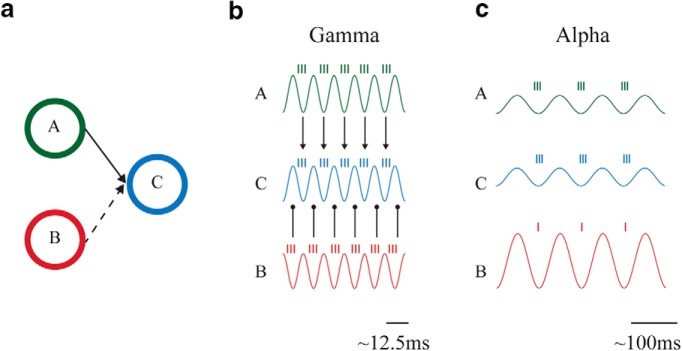
The communication through coherence (CTC) and gating through inhibition (GBI) hypotheses. ***a***, Two pools of neurons (A and B; e.g., in V1) are connected to a pool of neurons (C; e.g., V4). In this example, pool A communicates with C (solid line) while functional connectivity between B and C is suppressed (dashed line). ***b***, CTC. The waveforms represent oscillatory population activity (as measured in the LFP) in the three regions, whereas the small vertical lines represent spiking activity. The phase of the oscillatory activity modulates the excitability and thus spike timing. It is the phase relationship between the regions that determines the routing. The neurons in A and C oscillate in phase, whereas the neurons in B do not oscillate in phase with C. It has been proposed that this mechanism is implemented by gamma band oscillations (>30 Hz; Fries 2005) ***c***, GBI. The flow of information is controlled by an increase of alpha-band oscillations (∼10 Hz) which inhibits firing in pool B, and a decrease in alpha oscillations of neurons in A and C, allowing communication by release from inhibition (Jensen and Mazaheri 2010). It is the magnitude of the pulses of inhibition and thus the alpha power that controls the routing.

Consider two pools of neurons, A and B, that are connected to a third pool, C. As an example, this could be two subpopulations of neurons representing different spatial locations within V1 and project to a common subpopulation of V4 neurons that represent both spatial locations. When spatial attention is directed to the receptive fields (RFs) of neurons in pool A, the routing mechanism should favor the communication between A and C while preventing communication between B and C (Fig. 1*a*). How is the functional connectivity between A and C, but not B and C, established?

According to the CTC hypothesis, interregional communication is established when the oscillatory activity between these neuronal pools is coherent, i.e., they oscillate at the same frequency with a stable phase difference (Fries, 2005, 2009). This would allow the excitable phase of neurons in C to coincide with synaptic input from neurons in A. To block communication (the B-to-C pathway), the synaptic input from neurons in B arrives at the nonexcited phase of the neuron in C (Fig. 1*b*). Thus if B and C are not oscillating in phase synchrony, the communication is reduced. Brain regions have indeed been shown to phase-synchronize in the gamma band when attention is allocated (e.g., Womelsdorf et al., 2006, 2007; Bosman et al., 2012). How is the phase synchrony between regions A and C established? Fries and colleagues (Fries, 2009; Bastos et al., 2015) proposed that it is established by oscillations in neurons of pool A entraining neurons in C at the gamma frequency. This mechanism also implies that the phase synchronization among the neurons in A is stronger and potentially oscillates at a faster gamma frequency than in B (Fries, 2015). As a consequence, the neurons in C are entrained by A rather than B, thus dynamically strengthening the functional connectivity. This effect results in a mechanism that increases the impact of A on C while reducing the impact of B on C (Fries, 2009, 2015). It is not completely clear how B and C achieve asynchrony (Akam and Kullmann, 2014). As demonstrated in Bosman et al. (2012), coherence between B and C is reduced compared with coherence between A and C. The reduced coherence could be achieved by B and C fluctuating independently in the same frequency band, or by B and C oscillating at different frequencies (as shown in Bosman et al. [2012]). Yet, a possibility is that they oscillate at the same frequency but with a fixed phase difference (e.g., antiphase); however, this possibility seems at odds with the reduced coherence in the unattended pathway (Bosman et al., 2012).

According to the GBI hypothesis (Fig. 1*c*), the information flow between regions is established by actively inhibiting the pathway not required for the task. It has been proposed that alpha activity reflects regional-specific inhibition (Klimesch et al., 2007; Jensen and Mazaheri 2010; Foxe and Snyder 2011; Jensen et al., 2012). Alpha activity is associated with pulses of inhibition, i.e., the larger the alpha activity, the stronger the bouts of inhibition. This is consistent with findings from many experiments showing that alpha activity is high over task-irrelevant areas (Haegens et al., 2010; Snyder and Foxe 2010; Bonnefond and Jensen 2012; Capilla et al., 2014 but see Mo et al., 2011) or task-irrelevant groups of neurons within a brain area (van Kerkoerle et al., 2014). Furthermore, this increase has been shown to predict behavioral performance (Foxe et al., 1998; Thut et al., 2006; Meeuwissen et al., 2010; Haegens et al., 2011, 2012; Händel et al., 2011; Bonnefond and Jensen 2012, 2013; Payne et al., 2013; Myers et al., 2014). Considering Fig. 1*a*, gating would thus be reflected by alpha power increase in B and a decrease of alpha power in A and C. Furthermore, the alpha power decrease in A and C would allow for increased gamma power in these regions that could be involved in transmitting information. It is important to note that, in the latest version of the CTC framework (Fries, 2015), Fries also highlighted the potential role of alpha oscillations, in opposition to gamma oscillations, in preventing effective communication of local neuronal representations but also in holding these representations “on-stock” so that they can be flexibly used when needed.

Although both of these frameworks have strong explanatory value, they account for different findings in the literature. In particular, CTC in its current formulation does not address the issue of diverging routes where, e.g., one region is connected to two downstream regions. In this case, the entrainment by gamma oscillations does not provide a routing mechanism (but see Bastos et al., 2015; Fries, 2015). Several other challenges have been put forward to the CTC framework. One criticism is based on the finding that the gamma frequency is modulated by stimulus features such as contrast (Ray and Maunsell, 2010; Hadjipapas et al., 2015). This implies that different contrast levels that are part of a larger scene (e.g., a single object) are communicated at different frequencies, which might pose a problem for integrating this information in the converging visual hierarchy (Ray and Maunsell, 2010). However, Fries (2015) mentioned that the stimulus salience and level of attention to the subparts of a single object are often similar and thus result in similar gamma frequencies facilitating the integration in higher levels of hierarchy. However, a second criticism is based on an optogenic study that manipulated spike timing in the gamma and beta bands. Manipulating the temporal coordination of spiking activity did not influence behavior or transmission of spikes (Histed and Maunsell, 2014). Third, there is a debate as to whether the high-frequency activity generated by natural stimuli is dominated by band-limited oscillations in the gamma frequency range or nonoscillatory changes over a broad range of frequencies (Hermes et al., 2014b ; see “Existing evidence and predictions for communication by nested oscillations”). Finally, it has been argued based on results from a modeling study that, while entrainment might occur, communication is established already before coherence (Rolls et al., 2012).

Also the GTI framework is associated with several limitations. First, the phase modulation of neuronal firing by the alpha-band activity is not made explicit (Jensen et al., 2012, 2014). Second, GTI does not elaborate on the role of gamma-band activity for interareal communication, nor does it consider interregional phase synchrony in the alpha band as also being involved in interregional communication. Third, the strong emphasis on the alpha-band activity seems at odds with many nonhuman primate studies on attention in which modulations by alpha oscillations have only been recently reported, mainly via the use of laminar recordings (Haegens et al., 2011, 2015; Maier et al., 2011; Spaak et al., 2012).

At different levels of the cortical hierarchy, feedforward and feedback information needs be integrated (see Larkum, 2013 for one proposal). The GTI and CTC proposals are not fully explicit on the integration of feedforward and feedback at the microcircuit level (but see Lee et al., 2013 cited in Bastos et al., 2015 and Fries, 2015). Therefore it is crucial that these frameworks are unified and extended (e.g., by incorporating the phase-coding scheme), so that empirical studies can be specifically designed to test for the predictions derived from such a unified framework.

## A Unified Framework Based on Nested Oscillations

**Figure 2.**
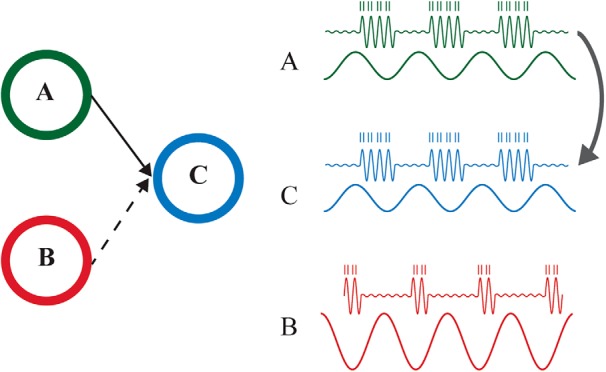
The new framework. The synchronization in the alpha-band establishes the functional connection between A and C. This allows for representational specific neuronal firing reflected by the gamma band activity to flow to region C. The blocking of communication between B and C is achieved by high alpha power in B and an asynchrony between B and C. Therefore both modulations in alpha-band power, as in gating by inhibition, and phase synchronization between the regions, as in CTC, are determining the routing of information between regions. Note that phase synchronization is assumed in the alpha band and the information transfer is reflect by gamma-band activity.

We here propose a unified framework which is based on the coupling of slow and fast oscillations (see also Lakatos et al., 2005; Schroeder and Lakatos, 2009; Florin and Baillet, 2015; Hyafil et al., 2015). In this framework (Fig. 2), we suggest that the information flow is established by neuronal synchronization at lower-frequencies in the theta (4–7 Hz), alpha (8–13 Hz), and beta (14–25 Hz) bands rather than in the gamma band. We will first develop the framework around the alpha band in the visual system (see “Communication based on nested oscillations could be a general mechanism throughout the brain” for a discussion about the role of beta oscillations in the visual system). This is motivated by the fact that there are a numerous empirical reports on alpha oscillations in the visual system in the context of experiments in which routing is manipulated using attention task. We will then discuss how the framework could generalize to other regions.

We consider here that alpha oscillations are associated with pulses of inhibition every ∼100 ms and as such can suppress neuronal activity locally as well as support interareal communication, through phase synchronization and release of inhibition (see also “Control of alpha oscillations in relation to cortical layers”). It is important to note that these mechanisms do not exclude complementary roles of alpha oscillations in other processes such as transmitting prior evidence to sensory areas (Sherman et al., 2016) or sampling (Busch and VanRullen, 2010; Song et al., 2014; VanRullen, 2016; see also “The role of saccades and slower rythms”).

We propose that, when neurons in pool A and C communicate, they oscillate coherently in the alpha band in conjunction with a decrease in alpha power. The decrease in alpha power creates longer windows of excitability in each cycle, i.e., longer duty-cycles (Jensen and Mazaheri, 2010), allowing for more information to be transferred between the synchronized regions. The blocking of communication between pool B and C is ensured by two complementary mechanisms: asynchrony between B and C preventing communication and stronger alpha power in B resulting in shorter duty-cycles. Asynchrony could mean that the regions are oscillating in antisynchrony, which could imply that they are still coherent. Another possibility is that they are not synchronized but fluctuating at different phases, albeit the frequencies are within the same range. In the latter case, the prevention of the transfer of information would be mainly implemented via an increase of alpha inhibition. Another possibility would be that pools B and C fluctuate at different frequencies.

Gamma oscillations are expected to be nested within alpha oscillations, i.e., they should occur only during the excitability phase of alpha oscillations. In pool A, the low magnitude of alpha oscillations allows for longer-duty cycles, i.e., longer time windows for the gamma activity during the excitability phase of the alpha cycle. As the excitable phase of the alpha oscillations will be aligned between the two relevant pools of neurons, gamma activity in A will be able to impact the neurons in C. This fast neuronal synchronization will have a strong impact on C owing to synaptic summation within the time window of a gamma cycle (Salinas and Sejnowski, 2001).

As a consequence, gamma oscillations in A and C will be correlated and possibly coherent. In pool B, the high magnitude of alpha oscillations will reduce the duty cycle, i.e., the gamma oscillations’ duration. In addition, the asynchrony of alpha oscillations in B and C will prevent gamma activity in B to drive cells in C. In short, a coupling between the phase of the alpha oscillations and gamma power could reflect the temporal coordination of information between regions.

The significance of alpha synchronization, specifically in a sensori-fronto-parietal network, in sensory processing was also emphasized by Palva and Palva (2007, 2011) and more recently by Siebenhühner et al. (2016). They proposed that cross-frequency phase coupling between alpha, beta, and gamma oscillations would allow the selection and maintenance of object representations during perception and working memory. They more specifically proposed that cross-frequency phase synchrony between the fronto-parietal network and the local gamma oscillations in sensory regions might underlie the incorporation of stimulus representations into the focus of attention. The current framework shares many similarities with their inspiring framework. The framework developed here is, however, very specific on how the modulation of (1) the local amplitude of alpha oscillations, (2) the interareal phase-alignment, and (3) the local interaction between phase alpha oscillations and the power (not the phase) of feedforward gamma oscillations is involved in the selective routing of information in cognitive networks. Furthermore, the current framework incorporates the phase coding scheme and discusses implementation of the model within the cortical layers. Finally, we attempt to generalize the model by considering that other slow rhythms could implement the specific interareal communication in other networks.

## Existing Evidence and Predictions for Communication by Nested Oscillations

Testing the proposed framework would require recordings from different regions in humans or nonhuman primates in the context of a task as, for instance, done by Saalmann et al. (2012). In that study, monkeys were cued to covertly attend to one of six locations after which a target array appeared. In the delay between cue and target, the allocation of covert attention was associated with an increase in coherence between V4 and temporo-occipital areas. Moreover, gamma coherence between V4 and TEO phase-locked to the alpha oscillations was observed. These results support our framework by demonstrating that alpha-band coherence is in control of the communication. While these findings provide first support for our framework, we will outline set of more specific predictions applied to the visual system in the following.

### Prediction 1: Alpha oscillations are a consequence of internal control, while gamma activity reflects feedforward communication; moreover, gamma activity is phase-locked to the alpha oscillations

The framework predicts that alpha oscillations set up the communication between relevant areas in a given task context. This idea implies that the phase and power of the alpha oscillations are under internal control. The gamma oscillations phase-modulated by the alpha oscillations will then reflect the information to be transferred in a feedforward manner.

Several recent articles have provided evidence in favor of slow frequency activity (alpha and beta oscillations) reflecting feedback control and gamma activity reflecting feedforward processing within the visual hierarchy (von Stein et al., 2000; van Kerkoerle et al., 2014; Bastos et al., 2015; Jensen et al., 2015; Michalareas et al., 2016; see also Arnal et al., 2011). Using Granger causality measures, they showed that alpha/beta oscillations in higher-order visual regions impacted lower-order regions during an attention task, while the reverse was observed for gamma oscillations. van Kerkoerle et al. (2014) further demonstrated that electrical stimulation of V1 induced an increase of gamma activity in V4, while stimulating V4 induced an increase of alpha oscillations in V1. The prediction regarding the control of alpha oscillations goes beyond feedback control, and we elaborate on many possibilities on where alpha is generated in “Control of alpha oscillations in relation to cortical layers.” It is important to note that gamma oscillations also can reflect feedback communication (Bastos et al., 2015; Michalareas et al., 2016), possibly controlled by alpha oscillations.

It has recently been shown that alpha and gamma activity interacts: gamma activity is phase-coupled to alpha oscillations during rest and during stimulus anticipation and processing in both monkeys and humans (Voytek et al., 2010; Spaak et al., 2012; Khan et al., 2013; Berman et al., 2014; Bonnefond and Jensen, 2015; Florin and Baillet, 2015). Two studies have demonstrated that the higher the alpha activity, the lower the gamma activity at a specific phase of alpha oscillations during rest in monkeys and during the retention period of a working memory task in humans. This is in line with alpha activity being associated with pulses of inhibition every ∼100 ms (Spaak et al., 2012; Bonnefond and Jensen, 2015). We propose that gamma oscillations, nested within slow oscillations, serve to segment neuronal representations in time. According to this framework, a neuronal representation is constituted by a distributed firing pattern constrained to a given gamma cycle (Lisman and Idiart, 1995). This allows for several items to be multiplexed over a gamma cycle (see “Exchange of phase encoded information”).

However, there is currently a debate about whether gamma activity reflects oscillations or whether it is a broadband phenomenon devoid of rhythmicity, in particular for >80 Hz oscillations (Lachaux et al., 2005; Brunet et al., 2014; Hermes et al., 2014b ; Buzsáki and Schomburg 2015; Ray and Maunsell 2015). The broadband activity (often called high-gamma activity or even epsilon when >80 Hz) is likely to reflect a hash of neuronal spiking rather than oscillations. However, it is possible that the 30- to 150-Hz activity is composed of both true oscillatory gamma activity (Brunet et al., 2014) and broadband multiunit activity (Manning et al., 2009; Ray and Maunsell, 2011). Possibly, the 80- to 150 Hz activity reflects the firing of neural populations, which is phase-locked to gamma oscillations at lower frequencies (30–80 Hz), as has been observed in the rat hippocampus (Belluscio et al., 2012).

This is an important issue, as the CTC framework articulates a mechanistic role for the phase of gamma oscillations such that the information can be transferred via interareal synchrony. The nature of activities observed in different gamma frequencies needs therefore to be further investigated using intracranial animal or human data.

In the present framework, the neuronal representation could contain both rhythmical activity (cf. e.g., “Exchange of phase encoded information”) or broadband activity devoid of rhythmicity, as the selectivity of the communication is subserved by an interareal synchronization at lower frequencies, i.e., theta, alpha, or beta.

Moreover, as pointed out above, the modulation of gamma frequency by different visual features (e.g., contrast) is difficult to reconcile with the CTC framework. This would result in different parts of an object being communicated at different frequencies in the visual hierarchy, preventing integration (but see Fries, 2015). In the present framework, the gamma activity generated by the different features of a stimulus would be represented at different phases of a single alpha cycle in a multiplexing manner, which then allows for integration. In future work, it will be interesting to investigate whether alpha oscillations indeed serve to group and integrate sensory input.

### Prediction 2: Alpha magnitude and interareal synchrony control the transfer of information carried by gamma oscillations

The framework assumes that alpha-band phase synchrony between A and C (see Fig. 2) allows stimulus-driven gamma oscillations, modulated by the phase of the alpha oscillations to be transferred from A to C. Specifically, we hypothesize that the synchrony of alpha oscillations between relevant areas predicts interareal correlation or coherence of gamma oscillations.

While the inter-areal coherence observed in the alpha and gamma bands during several cognitive processes in rats, monkeys, and humans (Womelsdorf et al., 2006; Pollok et al., 2007; Bastos et al., 2012, 2015; Grothe et al., 2012; Muller and Weisz, 2012; Popov et al., 2013; van Kerkoerle et al., 2014) supports the notion that phase synchronization reflects information exchange, the role of cross-frequency interactions needs to be explored in greater detail.

Our framework also predicts that the oscillatory dynamics can prevent the transfer of information from region B to C. This is achieved by strong alpha oscillations in B which are in asynchrony or antisynchrony with oscillations in C. This results in the hypotheses that (i) alpha power is strong in task-irrelevant areas (i.e., B) and (ii) there is a change in the phase relation between task-irrelevant (B) and downstream (C) regions. This might be reflected by antisynchrony or a decrease in synchrony (possibly due to a change in frequency in one of the pool). The power increase and synchrony decrease will be associated with less interareal power-correlation or coherence in the gamma band.

There is strong support for alpha magnitude increasing in task-irrelevant regions during attention and memory tasks (Foxe et al., 1998; Worden et al., 2000; Thut et al., 2006; Snyder and Foxe 2010; Banerjee et al., 2011; Bonnefond and Jensen, 2012; Payne et al., 2013). For instance, alpha oscillations have been shown to increase in the early visual regions in anticipation of a distractor in working memory tasks (Bonnefond and Jensen, 2012; Payne et al., 2013). With respect to the interregional phase relationship, antisynchrony (∼180° phase difference) between parietal and frontal areas during an oculomotor, delayed-match-to-sample task has been reported (Dotson et al., 2014); however, there are also findings demonstrating a decrease in synchronization between visual regions when spatial attention is directed away (e.g., Saalmann et al., 2012; Bastos et al., 2015).

Future investigations are required to identify when and where the mechanisms for preventing information transfer are at play.

### Prediction 3: To allow communication between two specific pools of neurons, alpha oscillations must be modulated locally on a fine spatial scale

The framework proposes that alpha oscillations are differently modulated in neuronal pools A and B (see Fig. 1*c*). This should be the case even if the stimuli processed by A and B are close to each other in retinotopic space. This results in the hypothesis that alpha oscillations must be modulated locally.

However, while it is often assumed that alpha oscillations in the visual system are modulated more globally (Thut et al., 2006), recent monkey and human Electrocorticography (EcoG) data provide promising evidence that alpha oscillations can be modulated locally even at the receptive field level. More precisely, these studies have shown that alpha oscillations increased (compared with baseline) in the surround area of the stimulated/attended receptive field in V1 (Harvey et al., 2013; van Kerkoerle et al., 2014). This does not preclude the need for alpha oscillations to be modulated more globally in the visual system in some situations, such as during working memory maintenance to protect against distractors (Bonnefond and Jensen, 2012) or during alertness (Sadaghiani and Kleinschmidt, 2016).

## Communication Based on Nested Oscillations Could Be a General Mechanism throughout the Brain

Thus far, we have focused on the coordinating role of alpha oscillations in the visual network. Further modulation of alpha activity related to functional inhibition has been reported in the language network in humans (Wang et al., 2012), in prefrontal regions in the monkey (Buschman et al., 2012; Engel, 2012; Jensen and Bonnefond, 2013; Welberg 2013), and even in the hippocampus (Staresina et al., 2016; but see below for a discussion regarding the hippocampus). However, similar functional roles may be played by oscillations at other frequencies, which have been shown to be prominent in other brain regions. For instance, there is strong evidence for the coupling in the theta band between the hippocampus and other regions such as prefrontal cortex, amygdala, and striatum (e.g., Seidenbecher et al., 2003; Benchenane et al., 2010; Kaplan et al., 2014; Staudigl and Hanslmayr, 2013; Backus et al., 2016). Importantly, when theta and alpha activity are observed in intracranial recordings across species, gamma power is typically found to be coupled to the phase of these oscillations (Canolty et al., 2006; Colgin et al., 2009; Canolty and Knight, 2010; Belluscio et al., 2012; Hermes et al., 2014a ; Jensen and Colgin, 2007; Voytek et al., 2010; Spaak et al., 2012). Thus, theta oscillations may play the same functional role as alpha oscillations in coordinating neuronal processing. Several studies also points to theta being inhibitory (Mehta et al., 2002). Given that theta oscillations in the monkey hippocampus have been found to overlap in frequency with alpha oscillations (Jutras et al., 2013), it remains an exciting possibility that the visual cortex and the hippocampus communicate via synchronization by means of these oscillations (Fell et al., 2011). However, it is debated whether the human theta rhythm is at ∼3 Hz or in a higher band (Watrous et al., 2013). In case it would be at 3Hz, the interactions between the visual cortex and hippocampus could then occur via cross-frequency coupling (Gu et al., 2015). The involvement of theta oscillations for coordinating the interactions between the striatum and the cortex during motor behavior in rats has been revealed by von Nicolai et al. (2014). In line with the current model, they further showed that the coordination of fast oscillations occurred via the coherent coupling of theta phase and high-frequency amplitude.

Beta-band oscillations might also play a role for coordinating the information flow by means of cross-frequency coupling, in e.g., the motor network (van Wijk et al., 2016). Moreover, some studies have reported attentional modulation of feedback-related beta oscillations in the visual system (e.g., Bastos et al., 2015; Kornblith et al., 2016). It remains to be explored what the functional differences between alpha and beta oscillations are. In particular, it will be important to determine whether the beta activity in some cases results from nonlinear addition of different alpha generators in different cortical layers (Jones et al., 2009). In line with this idea, laminar recordings have only revealed modulation of alpha oscillations in the visual system during attentional tasks (e.g., Bollimunta et al., 2011; van Kerkoerle et al., 2014), while EcoG recordings have revealed modulation of beta oscillations (Bastos et al., 2015).

## Exchange of Phase-Encoded Information

**Figure 3.**
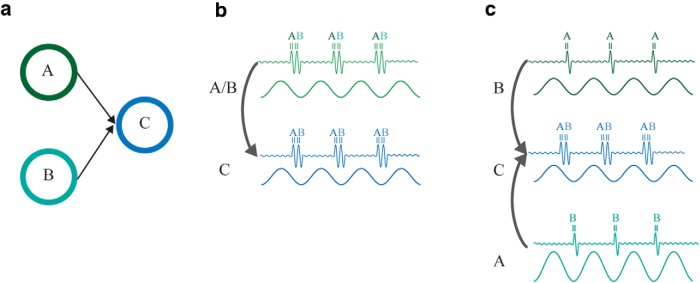
Exchange of phase coded information. ***a***, Two stimuli processed by two pools of neurons A and B, e.g., in V1. The pools both project to a pool of neurons C downstream in the hierarchy, e.g., in V4. Because of this bottleneck in the visual system, it is important that neurons coding for A and B in V1 are not activated simultaneously. For the information related to the two stimuli to be transferred from V1 to V4, we propose two mechanisms. ***b***, A single alpha generator in V1 controls for the timing of activation of neurons in pool A and B as reflected in the gamma band. The activation of the most excitable neurons, i.e., cells in pool A, overcomes the pulse of inhibition early in the alpha cycle followed by neurons in pool B (see Jensen et al. 2014 for details). The temporal organization is then transmitted to the pool of neurons in C. ***c***, Another possibility is that the magnitude of the alpha oscillations is modulated locally and is lower for one of the representations compared with the other. Because the alpha inhibition is lower for A, the respective neurons fire earlier than B. This temporal organization is then transmitted to C.

Extensive work in the rat hippocampus has demonstrated that different information is encoded at different phases of the theta cycle. In particular, when a rat traverses a place field, the phase of firing of the respective place cell advances with respect to theta phase (O’Keefe and Recce, 1993). Several mechanisms have been proposed for how such a phase-organized code might emerge (Jensen and Lisman, 1996; Burgess and O’Keefe, 2011; Lisman and Jensen, 2013) . In analogy, Jensen et al. (2014) recently proposed a model for how visual information might be encoded along the phase of the alpha cycle. In the model, competing visual representations are represented at different phase of the alpha cycle to resolve the bottleneck problem in the visual system. Due to the convergence in the hierarchy of the visual ventral stream, two stimuli (e.g., faces) might partly share the same neuronal representation in higher-order visual areas. Jensen et al. proposed that the processing of these two stimuli is segmented in time by being represented at different phases of the alpha cycle. The stronger the excitability of a given representation, the earlier it overcomes the inhibition as it ramps down within an alpha cycle. This creates a temporal code organized according to excitability (Jensen et al., 2014). As proposed for the hippocampus (Jensen, 2001; Colgin, 2011), the exchange of phase-encoded information can be achieved by phase-synchronizing the communicating networks (see Fig. 3).

Consider two representations associated with neurons in pool A and B. The excitability is stronger for A than for B (Fig. 3*a*). Jensen et al. (2014) proposed that the neuronal firing associated with each stimulus occurs at different phases of the alpha cycle. Moreover, A, B, and C would be synchronized in the alpha band and, as a consequence, this temporal organization would then be transmitted to C (Fig. 3*b*). A possibility Jensen et al. (2014) did not discuss is that the magnitude of the alpha oscillations in the two pools determines which stimulus is processed first (Fig. 3*c*). In particular, alpha power will be lower in pool A than in pool B, if the stimulus processed by the former is more relevant/salient. As a consequence, the gamma burst will occur earlier in A than in B due to the stronger alpha inhibition in the latter. Importantly, alpha oscillations are still expected to be synchronous between the three pools. To date, there is little empirical evidence demonstrating that alpha phase organizes neuronal coding. There is work in the δ, theta, and beta bands demonstrating phase-coding (Kayser et al., 2009; Voytek et al., 2015; Watrous et al., 2015). We call for future studies in which a phase-specific code is investigated in the visual system in the alpha band.

## Control of Alpha Oscillations in Relation to Cortical Layers

The framework we propose assumes that alpha oscillations are internally controlled in terms of phase and magnitude. We here discuss the mechanisms involved in the control. The control serves to phase-synchronize the oscillations between different regions and modulate the degree of pulsed inhibition to allocate computational resources. A number of studies have shown that alpha magnitude and phase can be modulated in anticipation of relevant or irrelevant stimuli (Foxe et al., 1998; Thut et al., 2006; Foxe and Snyder, 2011; Bonnefond and Jensen, 2012; Samaha et al., 2015; but see van Diepen et al., 2015), indicating that alpha oscillatory activity is indeed under internal control. In this section, we discuss two complementary mechanisms for this control, namely that alpha oscillations are controlled by neocortical feedback connections or by the thalamus. We will discuss this in the context of layer-specific computations.

### Feedback in Relation to Cortical Layers and Canonical Microcircuits

**Figure 4.**
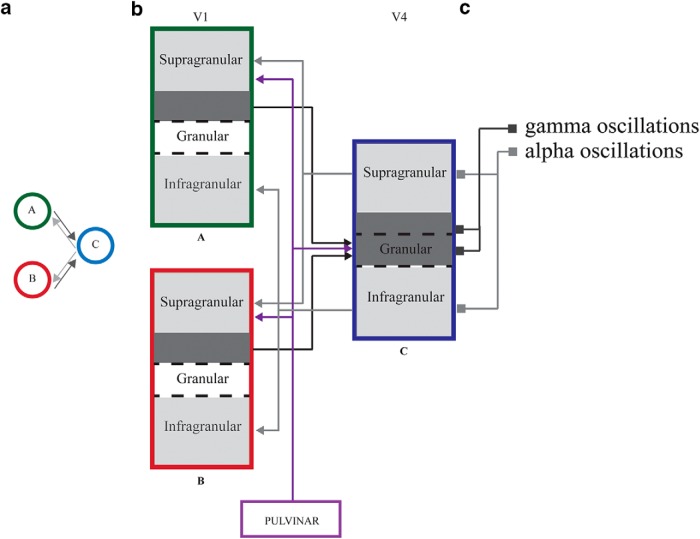
Converging feed-forward and diverging feedback pathways. ***a***, Pools of neurons A and B converge on a pool of neurons in C. Black arrows represent the converging feedforward pathway and the gray arrows represent the diverging feedback pathway. ***b***, Example in which two cortical columns in V1 (A and B) are connected to a column in V4 (C). Three layers are represented, the supragranular, the granular, and the infragranular. Dark and light grays represented in the layers are involved in the feedforward and feedback pathways, respectively. The layers associated with each pathway are inspired by Markov et al. (2014). The feedforward connections from the pulvinar are also indicated (purple arrows). ***c***, gamma and alpha oscillations have been shown to be prominent in the granular/supragranular and infragranular/supragranular layers, respectively.

Interestingly, the pools of neurons involved in the feedforward and feedback pathways are segregated in different cortical layers in the visual system (Markov et al., 2014). The cortical layers involved in feedforward and feedback differ according to the hierarchical distance between the connected brain regions (Barone et al., 2000; Markov et al., 2011, 2014). In the case of the connections between V1 and V4, the feedforward pathway originates in supragranular layers (L3B) in V1 and target granular layers (L4) and L3B in V4. The feedback pathway from V4 originates in the supragranular layers L3A and infragranular layers L6 and target supragranular layers L1–2/3A and infragranular layer L6, respectively, in V1 (Fig. 4). Interestingly, alpha activity has been observed mainly in both the supragranular and infragranular layers of a given area, with a stronger power in the latter (but see Haegens et al., 2015), while gamma activity has been shown to be prominent in the granular and supragranular layers (Bollimunta et al., 2008, 2011; Buffalo et al., 2011; Maier et al., 2011; Spaak et al., 2012; van Kerkoerle et al., 2014; Dougherty et al., 2015).

According to our model, supragranular and/or infragranular alpha oscillations should exercise an inhibitory phasic influence on the granular and/or supragranular gamma magnitude (see Spaak et al., 2012; Fig. 4*b*).

As shown in Fig. 4, the feedback is diverging when originating in V4 and projecting back to several V1 regions. This feedback needs to be selective, e.g., determining the alpha-phase synchronization from C to A, but not C to B. Moreover, local alpha magnitude in A and B should be distinct, with a higher alpha magnitude in B. We discuss below the putative role of different neocortical and subcortical regions in modulating the local change in alpha magnitude and the alpha synchronization between communicating areas.

It remains to be better understood how alpha and gamma oscillations are generated from a physiologic perspective and how their interaction is implemented at the level of the microcircuit. The mechanisms generating gamma oscillations have been extensively reviewed in Buzsáki and Wang (2012), but less is known about alpha oscillations. Alpha oscillations are thought to involve inhibitory neurons to set up pulses of inhibition every ∼100 ms. Somatostatin cells engaged via lateral connections (Zhang et al., 2014) or trans-laminar fast-spiking neurons engaged by layer 6 neurons (Olsen et al., 2012; Bortone et al., 2014) or even layer 5 pyramidal cells (Buchanan et al., 2012) are strong candidates, but further research is needed to evaluate their behavior during alpha oscillations. Layer 1 interneurons might also be involved as dendrites from layers 2/3A, 3B, and 5 reach this layer (Markov et al., 2014). Also, the role of lateral connections (Wang et al., 2000; Angelucci and Bullier, 2003; Tamura et al., 2004) and the role of the thalamus (da Silva et al., 1973; Lorincz et al., 2009; Vijayan and Kopell 2012) need to be investigated.

The examples considered here concern the visual system. It has been shown, however, that the laminar organization (e.g., cortical types can be granular, agranular, or dysgranular) and the connectivity between areas varies across networks (see e.g., Rempel-Clower and Barbas, 2000). It will be important to consider these anatomic, but also functional, heterogeneities to further determine whether the information is communicated by similar principles in these different networks.

### Regions Involved in the Control of Alpha

Several studies have investigated the influences of the fronto-parietal network, i.e., the frontal eye field (FEF) and the posterior parietal cortex, on activity of posterior regions (Szczepanski et al., 2010; Noudoost and Moore, 2011; Squire et al., 2013). The fronto-parietal network includes a number of areas that are retinotopically organized, and it is engaged during spatial attention, saccade planning, and other cognitive and perceptual operations (Saygin and Sereno, 2008; Silver and Kastner, 2009). The fronto-parietal network is directly and indirectly (through the pulvinar) connected to visual regions. The FEF and parietal cortex have both been shown to be associated with the control of alpha activity in posterior regions in humans (Capotosto et al., 2009; Marshall et al., 2015b ; Sauseng et al., 2011) potentially via the superior longitudinal fasciculus (Marshall et al., 2015a ). However, the role of the fronto-parietal network for controlling alpha phase remains to be elucidated (but see Sauseng et al., 2005).

Recently, Sadaghiani et al. (2016) further proposed that different cortical networks were involved in controlling alpha oscillations. More specifically, they proposed that a network including the dorsolateral prefrontal cortex, the rostrolateral prefrontal cortex, the posterior inferior parietal lobe, the paracingulate gyrus, and the mid-cingulate gyrus was involved in controlling long-range alpha-phase locking associated with adaptive control, while the dorsal attention network (including intraparietal sulci, frontal eye fields, and middle temporal complex) was associated with controlling the (dis-) engagement of regions via the control of local alpha amplitude and as such with implementing selective attention.

Subcortical regions might also play a key role in modulating alpha activity. For instance, the pulvinar is in a particularly well-suited anatomic position for controlling the communication between posterior neocortical areas, since it is connected to a wide range of areas in the visual hierarchy. More specifically, it is connected to neighboring cortical regions that are themselves directly connected to each other (Saalmann and Kastner, 2011; Saalmann et al., 2012). Here, we consider two ways in which the pulvinar might influence the synchronization in the alpha band between brain regions. (1) The pulvinar might modulate the feedback originating from higher-order regions as it targets layers 1–3) of the lower-order area. In line with this idea, Purushothaman et al., (2012) showed that electrical stimulation of pulvinar neurons in anesthetized prosimian primates resulted in boosting the firing of V1 neurons when stimuli were presented in the V1 neurons’ receptive fields, while it suppressed the neuronal activity when the stimuli were presented outside the receptive field. As such, the stimulation mimicked the effects of attention. Following the schema shown in Fig. 4, we suggest that the pulvinar serves to synchronize C and A by increasing the impact of the feedback connections arriving in L1–3. Likewise, the pulvinar might decrease the activity in B, reducing the synchrony between C and B. It is further possible that the pulvinar increases the magnitude of alpha oscillations in B. (2) The pulvinar might directly control the synchronization of alpha activity between two areas, as it is connected to supragranular layers (L1–3) of the lower-order area (e.g., V1) and granular layer (L4) of the higher-order area (e.g., V4; Saalmann and Kastner, 2011). Although alpha activity is thought to be particularly high in supragranular and infragranular layers, several papers have also revealed the presence of an alpha generator in L4 (Bollimunta et al., 2008, 2011; Haegens et al., 2015). It is thus possible that the pulvinar allows the synchronization between L1–3 in a lower-order region and L4, which receives the feedforward activity, in a higher-order region. In line with this idea, Saalmann et al. (2012) demonstrated, using a measure of Granger causality, that the pulvinar was driving the alpha-band synchronization between V4 and TEO when attention was allocated at the receptive field of the regions recorded. However, they did not observe an increase of the amplitude of alpha oscillations in these cortical areas when attention was directed away from it (Kastner, unpublished observations). Such amplitude change might occur only in V1. Therefore, they could not investigate the influence of the pulvinar on a change in alpha amplitude as suggested in the paragraph above. Interestingly, the pulvinar is also known to be connected to frontal areas (Saalmann and Kastner, 2011). It is therefore possible that part of the influence of frontal areas on the sensory cortex is mediated by the pulvinar. In summary, the mechanisms underlying the influences of the pulvinar on alpha oscillations in the different cortical areas remain to be understood. In addition to the pulvinar, interactions between the prefrontal cortex, the thalamic reticular nucleus (TRN), and the lateral geniculate nucleus might also be involved in setting up alpha-power increases in early visual regions. Recent articles have shown that the prefrontal cortex directly influences TRN activity, thereby controlling thalamic sensory gain during attention (Halassa et al., 2011; Wimmer et al., 2015).

Further investigations, such as exploring the task-specific laminar profiles of alpha oscillations, will be necessary to determine how the feedback activity from higher visual regions and the different cortical and subcortical regions influence the power and the phase of alpha activity across the visual network. In particular, it will be useful to determine how the phase synchrony (both synchrony and antisynchrony) is implemented. It is possible that the alpha oscillations observed in different layers have distinct roles for coordinating communication. For instance, alpha in supragranular layers might be involved in coordinating interareal communication over long distances, while alpha in infragranular layers might be involved in more local control of granular and supragranular gamma power. Indeed, it has been shown that the supragranular layers exhibit a spatially specific connectivity in both the feedforward and feedback pathways, while the infragranular layers exhibit a more diffuse connectivity (Markov et al., 2014). The more diffuse connectivity might be related to the role of alpha oscillations in the inhibition of all nonrelevant cortical columns in a rather unspecific way, while the spatially specific connectivity could be related to the communication of alpha activity within the relevant pools of neurons.

## The Role of Saccades and Slower Rhythms

In most electrophysiological studies in humans and animals on attention and visual perception, fixation is kept constant. However, in daily life, we make saccades several times per second. Furthermore, even when fixating, microsaccades at 3–4 Hz are apparent (Bosman et al., 2009; Lowet et al., 2016). In future work, it will be of great interest to investigate how saccade relates to coupled alpha and gamma oscillations. One intriguing possibility is that the (micro-)saccades are coordinated with alpha oscillations (Gaarder et al., 1966; Drewes and VanRullen, 2011). While spatial sampling could involve saccades, it could as well be implemented by rhythmic shifts of spatial attention at slow frequency (Landau and Fries, 2012; Fiebelkorn et al., 2013; Song et al., 2014; Landau et al., 2015; VanRullen, 2016).

In the latest version of the CTC, Fries (2015) further develops the idea that the cross-frequency coupling between theta and gamma oscillations implements visual attentional sampling (Bosman et al., 2009, 2012). The sampling role of theta oscillations in the visual system proposed by Fries (2015), which seems to be transmitted in the feedforward direction (van Kerkoerle et al. 2014; Bastos et al. 2015), is different from the role of alpha oscillations we propose. Specifically we suggest that the role of alpha oscillations is to implement interareal communication by modulating interareal phase synchronization and the local magnitude. However, recent results suggest that the theta and alpha rhythms could interact. Song et al. (2014) presented interesting results showing that the behavioral performances (in terms of reaction time) in an attention task (discrimination of a square or a circle) was modulated in the alpha range, but alternated between the cued and uncued side at a theta rhythm (3–4 Hz), i.e., the behavior exhibited a theta–alpha coupling.

Further work is needed to understand in which situations such slower rhythms are required and how they interact with the alpha and gamma oscillations in relation to (micro-)saccades. Finally, it is important to develop a model integrating the feedforward sweep that is evoked by saccades and microsaccades (Gaarder et al., 1966; Ito et al., 2013).

## Conclusion

In this article, we have proposed a framework for flexible communication between interconnected nodes in the brain based on the coupling between slow oscillations in the theta/alpha band and activity in the gamma band. Testing the framework will require integrating animal and human research to relate spiking to behavior from a mechanistic perspective. This will allow for elucidating how representational specific information is exchanged between brain regions. Finally, it needs to be understood how these cross-frequency interactions are internally controlled. Of particular interest is the involvement of the thalamus in coordinating oscillatory activity between regions.

## Synthesis

Reviewing Editor: Leonard Maler, University of Ottawa Decisions are customarily a result of the Reviewing Editor and the peer reviewers coming together and discussing their recommendations until a consensus is reached. When revisions are invited, a fact-based synthesis statement explaining their decision and outlining what is needed to prepare a revision will be listed below. The following reviewer(s) agreed to reveal their identity: Bradley Voytek, Nancy Kopell.

Editor:

Both reviewers find that this manuscript is a potentially relevant and useful contribution to an important field - the integration of two theories of the role of coherent oscillations in neural communication. There are, however, some serious difficulties; the second reviewer, in particular, has noted that some key recent papers were not cited and that the logic of the story was not entirely clear. The authors are encouraged to carefully address all the concerns of both reviewers. The revised manuscript will then be re-reviewed by both reviewers.

Reviewer #1

The manuscript, “Communication between brain areas based on nested oscillations,” aims at introducing a “unifying” account for the role of neural oscillations in coordinating information transfer between brain regions. It is an explicit attempt at unifying oscillatory phase-based and amplitude based models, namely Communication Through Coherence and Gating by Inhibition.

Overall the author(s) seem to be very knowledgeable about the state of the field; they provide an excellent overview and synthesis of ideas and make a strong case for their proposed unifying account. While I remain not entirely convinced, my opinion on that mature should not factor into the decision on whether or not to publish this piece.

My main hesitation is that there are a few places where it feels like the author(s) are either using terminology loosely, intentionally, or on accident. Specifically, there is some hand-waving regarding “gamma”. Within the CTC framework, gamma, in this case, is truly a band-limited oscillation, whose phase is meaningful. In another sense, there is “high gamma”, sometimes called “broadband activity”, which is not an oscillation at all. The author(s) do briefly acknowledge this when they cite Hermes's work, but I think a more careful perspective is needed. There is currently an argument between the Fries camp and the Hermes/Miller camp about “gamma”, and it's important for this review.

The Fries side is making phase-based claims, the Hermes/Miller side is more saying the broadband reflects population spiking (c.f., Manning and Kahana, J Neurosci, 2009). This is fine, as low-frequency-to-high-gamma coupling, as in phase-amplitude coupling (PAC) can still fit the Fries view. But it requires more than a simple glossing over.

Specific questions and concerns:

• The author(s) begin by talking about “low frequency (< 25 Hz) oscillations”, but then largely focus on alpha. 25 Hz is well within the beta range, which has much clearly physiological origins than the slower theta/alpha oscillations.

• I'm surprised the author(s) don't cite Palva & Palva, Trends in Neurosciences, 2007. That review covers a lot of similar territory as this manuscript.

• The opening paragraph is, I believe, the weakest in the paper. Where is the evidence for the “millions of bits” statement? And that “most information is irrelevant for the task at hand”? Are we always needed to be “on task”?

• Additionally, the opening paragraph states that there must be a mechanism for selection/prioritization of information, but this isn't really a selection/suppression paper, so why set it up this way?

• All figures need to have the populations labeled A/B/C! For example, Fig. 1b and 1c show different populations. While they're colored according to the population colors in 1a, I printed this out in black and white and was initially confused until I realized the critical information was denoted by color.

Reviewer #2

This paper proposes a unifying hypothesis between two influential theories about communication in the brain: “communication through coherence” and a gating by inhibition theory of the role of the alpha oscillation. Together they are used to suggest “a novel mechanism for selective routing based on cross-frequency coupling between slow oscillations in the alpha and gamma band.” I agree with most of the points of the paper, which also contains very useful references. But I have two major concerns about the paper.

The first concern is about certain key papers that were omitted in this review. One is by Pascal Fries, who just published (in Neuron) a 10-year update to his theory about CTC. This paper addresses previous critiques of the CTC (including those by Maunsell), and hence is highly relevant to the part of the current paper that deals with critiques of CTC. It also discusses nested oscillations, especially nesting of gamma in theta/alpha, an important part of the current paper. Another key omission is the body of work by Palva and Palva, notably their 2007 paper on “New vistas for alpha-frequency band oscillations”. A sentence from their abstract is “We propose that simultaneous alpha-, beta- (14-30 Hz) and gamma- (30-70 Hz) frequency band oscillations are required for unified cognitive operations, and hypothesize that cross-frequency phase synchrony between alpha, beta and gamma oscillations coordinates the selection and maintenance of neuronal object representations during working memory, perception and consciousness.” This text is very close to the thesis of the current paper, and this reviewer struggled to understand what the authors were trying to emphasize that offered a new point of view.

The second concern was related: The authors cited criticism of the both CTC and the theory that alpha plays a role in selective inhibition. At least some of those criticisms are valid, partly because theories tend to be too general when they are first formulated, and it takes time and work to find out the circumstances under which they are or aren't valid. The alpha rhythm, as an example, appears to come in many forms, and is likely to be involved in many functions, including sampling. I found it hard to sort out the opinions of the authors on which alphas were important for the theory they are espousing. For example, do they think that the alpha/theta associated with the pulvinar works by inhibiting non-attended areas? If so, my reading of the relevant authors (e.g., Saalman/ Kastner) is that they would disagree. If not, how is that work relevant to a theory connecting the gating by inhibition hypothesis of alpha and CTC?

The bottom line here is that I found it hard to follow the logic of the story. Partly because of that, it was hard for me to tell what was truly novel. This may be correctable with revision. In general, I think it will be a useful review.

Other points/details:

1. In figure 2, there is asynchrony in alpha to prevent routing. What determines synchrony or asynchrony?

2. Prediction 1: Can the alpha be coming from sensory thalamus? If so, is that feedback?

3. As mentioned above, there appear to be many different kinds of alpha. Is the one being emphasized coming from the pulvinar? Are all forms of alpha participating?

4. Gamma is not always feed-forward; this is said in the relevant Fries paper, but most people only get the punchline that is (mostly) true.

5. I believe the debate over whether gamma reflects broadband phenomena rather than rhythms is only for the high frequency component of “gamma” (sometimes called epsilon), that is over 90 or 100 Hz.

6. I (and others) agree with the authors that cross-frequency coupling is likely to be very important in integration. I was hoping the authors of this paper would say something about how such integration comes about, especially if different inputs are appearing in different phases of the alpha.

7. In Prediction 2, the authors suggest that alpha controls the transfer of information via phase synchrony or not. Are they suggesting that there are no situation in which alpha produces its gating effect by a “bath” of inhibition, unrelated to phase? I believe that their prediction is likely to be correct in some instances, and it would be good to be clearer about what such circumstances might be.

8. The prediction that alpha can be modulated locally gets at the issue of multiple kinds of alpha. Is it possible that there are some that are more global than others?

9. The discussion of the role of layers seems to be based on the work from the Kennedy lab. Another major contributor to the relevant anatomy is Barbas. Do they agree on the issues discussed in the current paper?
